# Validity and reliability of a Hausa language questionnaire assessing information, motivation and Behavioural skills for malaria prevention during pregnancy

**DOI:** 10.1186/s12889-020-08513-y

**Published:** 2020-03-24

**Authors:** Ahmed Dahiru Balami, Salmiah Md. Said, Nor’afiah Mohd Zulkefli, Norsa’adah Bachok, Bala Muhammad Audu

**Affiliations:** 1 Department of Disease Control and Elimination, Medical Research Council The Gambia at London School of Hygiene and Tropical Medicine, Fajara, The Gambia; 2 Department of Community Health, Faculty of Medicine and Health Sciences, Universiti Putra Malaysia, Selangor Darul Ehsan, Malaysia; 3 Unit of Biostatistics and Research Methodology, Universiti Sains Malaysia, Kelantan, Malaysia; 4 Department of Obstetrics and Gynaecology, University of Maiduguri, Maiduguri, Nigeria

**Keywords:** Questionnaire, Hausa, Malaria in pregnancy, Information-motivation-behavioural skills, Validation

## Abstract

**Background:**

Many studies on malaria knowledge, attitude and practice among pregnant women have been conducted in Hausa speaking communities in Nigeria. Despite this, no standard and uniform instrument for assessing this important public health problem has been developed in the Hausa language, even though it is widely spoken. The aim of this study was to develop and validate a questionnaire in Hausa language assessing information, motivation, and behavioural skills for malaria prevention during pregnancy.

**Methods:**

The questionnaire was first developed in English language, and then assessed for its contents by a team of experts. It was then forwardly translated to Hausa, and backwardly translated again to English by independent language experts. These two English versions were then compared by a Public Health expert, following which the questionnaire was administered to 190 Hausa speaking antenatal care attendees. Exploratory factor analysis was performed on the data collected. Sixty three out of the 190 respondents were invited after 2 weeks to answer the same questionnaire, following which reliability tests were performed.

**Results:**

The questionnaire showed good internal consistency, with Cronbach’s alpha values of 0.859, 0.890 and 0.773 for information, motivation and behavioural skills constructs respectively. The motivation and behavioural skills constructs were able to delineate their items into three and two sub-sections respectively. The factor loadings for the two constructs ranged from 0.610 to 0.965. As for test retest reliability, the Krippendorff’s alpha values for the items of the motivation section ranged from 0.941 to 0.996; that for behavioural skills ranged from 0.810 to 0.953, while for frequency of ITN use, it was 0.988. The Cohen’s kappa values for the information section ranged from 0.689–0.974, except the item for ‘fever’ (*zazzabi*) which was 0.382, and was as such reworded to a simpler terminology ‘hotness of the body’ (*zafin jiki*).

**Conclusions:**

The Hausa language IMB questionnaire on malaria in pregnancy demonstrated good validity, and a high level of reliability. It is as such recommended for use among Hausa speaking communities to ensure uniformity and objectivity.

## Background

Malaria remains a public health problem in Nigeria, as it contributed the largest percent of cases (27%) to its global incidence in the year 2016 [1]. Malaria had also been reported to account for 11% of maternal mortality [2]. Despite the World Health Organization’s recommendations (WHO) for pregnant women in sub-Saharan Africa to always sleep under an insecticide-treated net (ITN) and take at least two doses of intermittent preventive treatment during their pregnancy (IPTp) [3], the level of compliance to these preventive measures has been very low among pregnant women in Nigeria [4]. Many studies have been conducted on knowledge, attitude and practice of pregnant women towards malaria, ITN, and IPTp, in Hausa speaking communities [5–11]. Hausa language is widely spoken not only in Nigeria, but also in many other African countries like Niger Republic, Ghana and Sudan, with an estimated 50 to 60 million people who understand the language to various degrees [12]. There exists the need to develop a valid uniform assessment tool in this widely spoken language, to enable uniform assessment by researchers, and facilitate the efficient monitoring of progress of public health interventions in that regard. Developing the instrument based on a health theory is likely to allow for a more thorough and systematic assessment of the health behaviour in question. The Protection Motivation Theory, while laying much emphasis on motivation, fails to identify other environmental and cognitive factors that can affect attitude change [13]. The information-motivation-behaviourial skills (IMB) theory was first developed to explain HIV preventive behaviours among college students [14]. This theory comprises of three components which are information about the health behaviours, motivation to carry out such behaviours, and the requisite skills for performing such behaviours [14]. The aim of this study was to develop and validate a questionnaire in Hausa language assessing information, motivation, and behavioural skills for malaria prevention during pregnancy.

## Methods

### Questionnaire development

The questionnaire comprises of four sections which are the information, motivation, behavioural skills and behaviour sections. For the information section, its items were adapted from the knowledge sections of some previous studies [6, 11, 15]. Items of the motivation and behavioural skills sections were developed from modifications of the relevant sections of the IMB questionnaire on diabetes mellitus self-care by Osborn et al. [16]. The questionnaire was first developed in English language, after which it was translated through the process of translation and adaptation of instruments outlined by the WHO [17].

It was at first forwardly translated to Hausa language by a senior University academic staff of the Hausa Language Department. This translation was then assessed by a native Hausa Public Health specialist, and then backwardly translated into English by a different translator of the same qualification. The two English versions (original and back-translated) were then compared by another Public Health specialist. This was followed by questionnaire testing.

### Questionnaire structure

#### Section A

This section had a total of 45 questions assessing the different domains of knowledge on malaria in pregnancy, which were: transmission, symptoms, complications, and prevention of malaria during pregnancy. There were three options for each question: ‘Yes’, ‘No’, and ‘I don’t know’. The total maximum obtainable information score was 45 points, while the minimum obtainable score was zero (0) points.

#### Section B

This section assessed participants’ level of motivation for sleeping under an ITN and taking IPTp. It had a total of 12 items, and comprised of two sub-sections (one on personal motivation, and the other on social motivation). The first four items on personal motivation asked of the participants’ perception of the level of goodness or otherwise of practicing those malaria preventive measures. These questions had response options on a five-point Likert scale thus: ‘very bad’, ‘somewhat bad’, ‘neither bad nor good’, ‘somewhat good’ and ‘very good’, which were scored 1, 2, 3, 4, and 5 points respectively. The next four questions on personal motivation assessed participants’ perception of the level of pleasantness or otherwise of practicing these preventive measures. These also had response options on a five-point Likert scale thus: ‘very unpleasant’, ‘somewhat unpleasant’, ‘neither unpleasant nor pleasant’, ‘somewhat pleasant’ and ‘very pleasant’, which were scored 1, 2, 3, 4, and 5 points respectively.

There were four questions on social motivation, which assessed how truly or not, their significant others thought they should comply with those malaria preventive measures. This section had response options on a six-point Likert scale thus: ‘very untrue’, ‘mostly untrue’, ‘untrue’, ‘true’, ‘mostly true’, and ‘very true’, which were scored 1, 2, 3, 4, 5, and 6 respectively. The total maximum obtainable motivation score was 64 points, while the minimum obtainable score was 12 points.

#### Section C

This section assessed participants’ levels of behavioural skills. It had a total of seven items and two sub-sections. The first sub-section had three items which assessed how hard or easy it was to comply with ITN and IPTp. Responses to this section were on a four-point Likert scale, thus: ‘very hard’, ‘hard’, ‘easy’, and ‘very easy’, which were scored 1, 2, 3, and 4 respectively. The second sub-section which assessed the level of effectiveness with which the participants could execute certain tasks relating to ITN use, had four items. This section had responses on a four-point Likert scale too, thus: ‘very ineffectively’, ‘ineffectively’, ‘effectively’ and ‘very effectively’, which were scored 1, 2, 3, and 4 respectively. The total maximum obtainable behavioural skills score was 28 points, while the minimum obtainable score was 7 points.

#### Section D

This section assessed their frequency of ITN use during pregnancy, that is, the number of days in a week in which they slept under an ITN. Frequency of ITN use was categorized as: Never, Seldom (once to twice weekly), Sometimes (thrice to 4 times a week), Often (5–6 times a week) and Almost always. These categories were scored as: 1, 2, 3, 4 and 5 respectively. This section also asked whether or not they had received any IPT, and the number of doses they had received.

#### Ethics approval and consent to participate

Ethical approval was obtained to carry out the research, from the Ethics Committee for Research Involving Human Subjects of the Universiti Putra Malaysia (UPM) (UPM/TNCPI/RMC/1.4.18.2 (JKEUPM). Permission was also obtained from the Ethics Committee of the State Specialist Hospital, Maiduguri (SSH/GEN/64/Vol.1). All the respondents were first taken through the respondent’s information sheet in Hausa language, after which informed verbal consent was obtained from them. This was due to the low literacy rates in the study location [18], and it had been approved by the JKEUPM.

#### Questionnaire testing

This was done in stages thus: content validity by experts, face validity by 20 pregnant women, test of construct validity by on different pregnant women, and finally test of reliability by 63 out of the 190 initial respondents.

#### Content validity

Content validity was assessed using an expert group [19] who went through the questionnaire to ensure that the wordings of its items were clear, and that they represent their content domain. The assessment team comprised of three Public Health specialists and an Obstetrics and Gynaecology specialist.

#### Face validity

Twenty antenatal care attendees were conveniently selected from a secondary-level health centre in Maiduguri, north-eastern Nigeria, to assess the questionnaire. The criteria for selection was to be a fluent Hausa speaker, and also be at their first antenatal visit for their index pregnancy. The questionnaires were administered to them by interviewers, following which they were asked to assess each section of the questionnaire, based on order of its questions, language clarity, and whether the questions under each construct appropriately measured the respective constructs. Order of questions was scored as Good order, Average order, or Poor order; language clarity was scored as Clear, Average or Confusing; while appropriateness of construct measurement was scored as Good, Average, or Poor.

#### Construct validity and reliability

A further cross-sectional study was conducted at the same antenatal clinic, a week after the face-validity study. A hundred and ninety respondents were conveniently selected using the same criteria as for face validity. They were similarly made to complete the questionnaire, and the data obtained was then analysed in IBM SPSS version 22. Internal consistency was measured using the Cronbach’s alpha. The motivation and behavioural skills constructs of the questionnaire were subjected to an exploratory factor analysis (EFA) to determine how properly the items of each of their respective sub-sections correctly fitted. The assumptions for conducting an EFA had been met, as the data for these two constructs were collected on an interval scale, and there were also positive correlations between all the items. Items with factor loadings less than 0.3 were suppressed, and for the rotation, the oblique method (promax) was chosen due to some high correlations among some items.

Two weeks after the first questionnaire administration, it was re-administered to 63, out of the 190 respondents. These 63 respondents were randomly selected from the complete list of the initial 190 respondents using the random function in Microsoft Excel 2013. The Cohen’s kappa was measured to determine the reliability between the answers at first and second administration, for items of the information section, since the responses were in a nominal form. For the motivation, behavioural skills and frequency of ITN use sections, the Krippendorff’s alpha were measured to determine reliability.

## Results

The results of face validity by the 20 respondents are presented in Table 1. For the information section, 85% rated the order of its questions as good, 95% rated its language clarity as clear, while 75% rated its appropriateness for its construct as good. For the motivation section, 75% rated the order of its questions as good, while 80% rated its language clarity as clear, and its appropriateness for its construct as good. For behavioural skills, 80% rated the order of its questions as good, 85% rated its language clarity as clear, while 80% rated its appropriateness for its construct as good. All other ratings given were average, with none of the ratings given as poor.

**Table 1:** Face validity results (*N* = 20)

**KNOWLEDGE**		
**Order of questions**	**Freq. (%)**	
Good order	17	(85)
Average	3	(15)
Poor order	0	(00)
Total	20	(100)
**Language clarity**	**Freq. (%)**	
Clear	19	(95)
Average	1	(5)
Confusing	0	(00)
Total	20	(100)
**Appropriately measures level of knowledge**	**Freq. (%)**	
Good	15	(75)
Average	5	(25)
Poor	0	(00)
Total	20	(100)
**MOTIVATION**		
**Order of questions**	**Freq. (%)**	
Good order	15	(75)
Average	5	(25)
Poor order	0	(00)
Total	20	(100)
**Language clarity**	**Freq. (%)**	
Clear	16	(80)
Average	4	(20)
Confusing	0	(00)
Total	20	(100)
**Appropriately measures level of motivation**	**Freq. (%)**	
Good	16	(80)
Average	4	(20)
Poor	0	(00)
Total	20	(100)
**BEHAVIOURAL SKILLS**		
**Order of questions**	**Freq. (%)**	
Good order	16	(80)
Average	4	(20)
Poor order	0	(00)
Total	20	(100)
**Language clarity**	**Freq. (%)**	
Clear	17	(85)
Average	3	(15)
Confusing	0	(00)
Total	20	(100)
**Appropriately measures level of behavioural skills**	**Freq. (%)**	
Good	16	(80)
Average	4	(20)
Poor	0	(00)
Total	20	(100)

For the subsequent questionnaire evaluation, the ages of the 190 respondents ranged from 17 to 45 years, with mean (SD) of 25.4 (5.5) years. Most of them were married in a monogamous setting (75.3%), around a third had some form of employment (32.1%), while 84.2% were multigravidae (Table 2).

**Table 2:** Respondents’ characteristics (*N* = 190)

Factor	Frequency (n)	Percentage (%)
**Age**		
< 20 years	25	13.2
≥ 20 years	165	86.8
Total	190	100.0
**Ethnicity**		
Kanuri	60	31.6
Hausa	46	24.2
Babur	19	10.0
Shuwa	16	8.4
Marghi	12	6.3
Fulani	14	7.4
Others	23	12.1
Total	190	100.0
**Family type**		
Monogamy	143	75.3
Polygamy	47	24.7
Total	190	100.0
**Education**		
None	74	38.9
Primary	32	16.8
Secondary	61	32.1
Tertiary	23	12.1
Total	190	100.0
**Occupation status**		
Employed	61	32.1
Not employed	129	67.9
Total	190	100.0
**Type of residence**		
Permanent resident	155	81.6
Internally displaced	35	18.4
Total	190	100.0
**Gravidity**		
Primgravida	30	15.8
Multigravida	91	47.9
Grandmultigravida	69	36.3
Total		

The Cronbach’s alpha results for these sections ranged from 0.773 to 0.889 as presented in Table 3. For motivation, enough items were predicted by each factor, as evidenced by Kaiser-Meyer-Olkin (KMO) of 0.840. The variables were correlated enough, as evidenced by a significant Bartlett’s test of Sphericity (< 0.001). Rotation sum of square factor loadings indicated that 69% of the total variance was being explained by the three significant factors. For behavioural skills, enough items were also predicted by each factor, as evidenced by KMO of 0.785. The Bartlett’s test of Sphericity was also significant (< 0.001). Rotation sum of square factor loadings indicate that 60% of the total variance was being explained by the two significant factors.

**Table 3:** Summary of Cronbach’s alpha results (*N* = 190)

Section	No. of items	Cronbach’s alpha
Knowledge	46	0.859
Motivation I (Question 1 to 8)	8	0.872
Motivation II (Question 9 to 12)	4	0.889
Behavioural skills	7	0.773

The initial factor loadings for the factor analysis of motivation are presented in Table 4. The motivation construct was able to delineate its items into three main categories, however Mot7 and Mot8 were not able to gauge level of pleasantness, but rather had high factor loadings for level of goodness. Mot1 also had moderate factor loading for level of pleasantness. Since both level of goodness and level of pleasantness were measuring personal motivation, the two items (Mot7 and Mot8) were dropped, since items with the same wordings appeared in the ‘level of goodness section’ (Mot3 and Mot4). The final factor loadings after the two items were dropped is presented in Table 5. There was still some moderate cross loading for Mot1, although this was a little less than the values in the initial analysis when Mot7 and Mot8 were included. The behavioural skills construct was able to delineate its items into two main categories with no significant cross loading of items (Table 6).

**Table 4:** Factor loadings based on factor analysis for the motivation construct (all items retained)

	Item summary	Subscale		
		Goodness	Trueness	Pleasantness
**a.**	***Don Allah a gaya mana yaya kyaun ko rashin kyaun waɗannan game da lafiyarki***			
Mot1	Rinƙa kwana a cikin gidan sauro mai feshin magani	0.542		0.370
Mot2	Rinƙa kwana akai-akai fiye da da a cikin gidan sauro mai feshin magani	0.555		
Mot3	Rinƙa shan maganin kariya daga cutar malariya da aka ba ni yayin goyon ciki	0.889		
Mot4	Rinƙa shan dukkan magungunan kariya daga cutar malariya da aka bani ko da ina jin lafiyata ƙalau	0.912		
**b.**	***Don Allah a gaya mana yaya daɗi ko rashin daɗin waɗannan halayen a gareki***			
Mot5	Rinƙa kwana a cikin gidan sauro mai feshin magani			0.912
Mot6	Rinƙa kwana akai-akai fiye da da a cikin gidan sauro mai feshin magani			0.932
Mot7	Rinƙa shan maganin kariya daga cutar malariya da aka ba ni yayin goyon ciki	0.441		0.397
Mot8	Rinƙa shan dukkan magungunan kariya daga cutar malariya da aka bani ko da ina jin lafiyata ƙalau	0.569		
**c.**	***Mutanen da ke da muhimmanci a gare ni suna tsammanin yakamata in …***			
Mot9	Rinƙa kwana a cikin gidan sauro mai feshin magani		0.760	
Mot10	Rinƙa kwana akai-akai fiye da da a cikin gidan sauro mai feshin magani		0.821	
Mot11	Rinƙa shan maganin kariya daga cutar malariya da aka ba ni yayin goyon ciki		0.953	
Mot12	Rinƙa shan maganin kariya daga cutar malariya da aka bani ko da ina jin lafiyata ƙalau		0.870

**Table 5:** Factor loadings based on factor analysis for the motivation construct (two items deleted)

	Item summary	Subscale		
		Goodness	Trueness	Pleasantness
**a.**	***Don Allah a gaya mana yaya kyaun ko rashin kyaun waɗannan game da lafiyarki***			
Mot1	Rinƙa kwana a cikin gidan sauro mai feshin magani	0.640		0.354
Mot2	Rinƙa kwana akai-akai fiye da da a cikin gidan sauro mai feshin magani	0.651		
Mot3	Rinƙa shan maganin kariya daga cutar malariya da aka ba ni yayin goyon ciki	0.897		
Mot4	Rinƙa shan dukkan magungunan kariya daga cutar malariya da aka bani ko da ina jin lafiyata ƙalau	0.878		
**b.**	***Don Allah a gaya mana yaya daɗi ko rashin daɗin waɗannan halayen a gareki***			
Mot5	Rinƙa kwana a cikin gidan sauro mai feshin magani			0.904
Mot6	Rinƙa kwana akai-akai fiye da da a cikin gidan sauro mai feshin magani			0.936
**c.**	***Mutanen da ke da muhimmanci a gare ni suna tsammanin yakamata in …***			
Mot9	Rinƙa kwana a cikin gidan sauro mai feshin magani		0.734	
Mot10	Rinƙa kwana akai-akai fiye da da a cikin gidan sauro mai feshin magani		0.794	
Mot11	Rinƙa shan maganin kariya daga cutar malariya da aka ba ni yayin goyon ciki		0.965	
Mot12	Rinƙa shan maganin kariya daga cutar malariya da aka bani ko da ina jin lafiyata ƙalau		0.880

**Table 6:** Factor loadings based on factor analysis for the behavioural skills construct

	Item summary	Subscale	
		Easiness	Effectiveness
**a.**	***A halin yanzu yaya wahalar ko sauƙin yadda zaki iya …***.		
BSkills1	Kwana a cikin gidan sauro mai feshin magani kowace rana?		0.610
BSkills2	Shanye dukkan maganin kariya daga zazzaɓin cizon sauro lokacin goyon ciki?		0.847
BSkills3	Shanye dukkan maganin kariya daga zazzaɓin cizon sauro ko da kikan ji ba daɗi?		0.756
**b.**	***A halin yanzu yaya ƙwarewa ko rashin ƙwarewarki wajan … .***		
BSkills4	Rataya gidan sauro daidai?	0.699	
BSkills5	Dubawa ko gyara huji da yagewar gidan sauro mai feshin magani?	0.779	
BSkills6	Ƙara adadin kwanaki a sati da kike kwana a cikin gidan sauro a halin yanzu?	0.678	
BSkills7	Shawo kan wasu don su goyi bayan kwana da kike a cikin gidan sauro?	0.884	

Table 7 presents the socio-demographic characteristics of the retest sample (*N* = 63) and the remaining sample (*N* = 127). Both groups were similar on all factors except employment status, for which the remaining sample had a higher proportion of unemployed persons compared to the retest group (*χ*^2^ = 3.939, *df* = 1, *p* = 0.049).

**Table 7:** Comparison of socio-demographic characteristics of test and re-test samples

Variables	Group				***χ***^***2***^	***df***	***p***
	Test sample Freq. (%) n = 127		Retest sample Freq. (%) n = 63				
**Age group**					0.421	1	0.516
Less than 20	14	(11.0)	9	(14.3)			
20 years and above	113	(89.0)	54	(85.7)			
**Ethnicity**					2.707	6	0.845
Kanuri	41	(32.3)	20	(31.7)			
Hausa	30	(23.6)	18	(28.6)			
Babur	13	(10.2)	4	(6.3)			
Shuwa	10	(7.9)	6	(9.5)			
Marghi	9	(7.1)	2	(3.2)			
Fulani	9	(7.1)	6	(9.5)			
Others	15	11.8)	7	(11.1)			
**Family type**					0.852	1	0.356
Monogamy	93	(73.2)	50	(79.4)			
Polygamy	34	(26.8)	13	(20.6)			
**Type of residence**					0.407	1	0.523
Permanent resident	102	(80.3)	53	(84.1)			
IDP	25	(19.7)	10	(15.9)			
**Education level**					1.295	3	0.730
None	52	(40.9)	21	(33.3)			
Primary	20	(15.7)	11	(17.5)			
Secondary	38	(29.9)	23	(36.5)			
Tertiary	17	(13.4)	8	(12.7)			
**Occupational status**					3.939	1	0.049
Not employed	92	(72.4)	36	(58.1)			
Employed	35	(27.6)	26	(41.9)			
**Type of residence**					0.407	1	0.523
Permanent resident	102	(80.3)	53	(84.1)			
IDP	25	(19.7)	10	(15.9)			
**Gravidity**					0.767	2	0.681
Primgravida	18	(14.2)	12	(19.0)			
Multigravida	63	(49.6)	30	(47.6)			
Grandmultigravida	46	(36.2)	21	(33.3)			

The Cohen’s kappa reliability test for ‘Info6’ was 0.382, while those of the other items of the information section ranged from 0.689 to 0.974 as shown in Table 8. The Krippendorff’s alpha values for the items of the motivation section ranged from 0.941 to 0.996, while that for the behavioural skills section ranged from 0.810 to 0.953 (Table 9). For frequency of ITN use, it was 0.988.

**Table 8:** Summary of test retest results for knowledge

SNo	Item	Cohen’s kappa
	***Ta yaya ake kamuwa da malariya?***	
Info1	Cizon sauro	(no variance)
Info2	Jiƙewa da ruwan sama	0.803
Info3	Sauyin yanayi	0.858
Info4	Cin wasu irin abinci	0.974
Info5	Aikin wahala a rana	0.817
	***Mene ne alamun cutar malariya?***	
Info6	**Zazzabi** later reworded to **Zafin jiki**	0.382
Info7	Karkarwa	0.705
Info8	Ciwon kai	0.821
Info9	Ciwon gaɓoɓi	0.740
Info10	Rashin son cin abinci	0.826
Info11	Jin bani da lafiya	0.850
Info12	Ɗacin baki	0.806
Info13	Jin amai	0.689
Info14	Yin amai	0.858
Info15	Jin kamar lafiya ta ƙalau	0.868
Info16	Shin sauron da ke yaɗa cutar malariya na iya cizo da rana?	0.862
Info17	Shin goyon ciki na iya ƙara kawo kamuwa da cutar malariya?	0.877
Info18	Shin cutar malariya na iya cutar da mai goyon ciki?	0.859
Info19	Shin cutar malariya na iya cutar da ɗan tayin ciki?	0.858
	***Wace irin illa malariya kan iya jawowa lokacin goyon ciki?***	
Info20	Tana iya sa mace mai ciki ta rasa isasshen jinni	0.875
Info21	Yin ɓari	0.850
Info22	Haihuwa ba lokacin da ya dace ba	0.900
Info23	Haddasa haihuwar ɗa/‘ya mai ƙarancin nauyi	0.865
Info24	Mutuwar uwa	0.839
Info25	Mutuwar ɗan tayi	0.841
Info26	Kina da masaniyar gidan sauron da ke ɗauke da feshin maganin sauro?	0.945
	***Me ake yi da gidan sauron da ke ɗauke da feshin maganin sauro?***	
Info27	Kawar da sauro	0.710
Info28	Kawar da ɓeraye	0.834
Info29	Gidan sauro mai feshin magani ya fi wanda ba feshin magani	0.868
Info30	Feshin maganin gidan sauron kan iya zamowa haɗari gare ni muddin na kwanta a cikinsa	0.778
	***Bayan tsawon wane lokaci ya kamata a wanke gidan sauro mai feshin magani?***	
Info31	Bayan wata 1	0.897
Info32	Bayan wata 3	0.903
Info33	Bayan wata 6	0.820
	***Da me ya kamata a wanke gidan sauro mai feshin maganin sauro?***	
Info34	Ruwa da sabulu	0.890
Info35	Ruwa da omo	0.818
	***A ina ya kamata a shanya gidan sauro mai feshin magani?***	
Info36	A inuwa	0.844
Info37	A rana	0.885
Info38	Kina da masaniya akan maganin da ake bayarwa na kariya lokacin goyon ciki?	0.913
	***Wane irin magani ake bayarwa don kariya daga cutar malariya lokacin goyon ciki?***	
Info39	Chloroquine	0.927
Info40	Fansidar	0.921
	***Nawa ne adadin kwayoyin maganin kariya daga cutar malariya da ake bayarwa kowane lokaci ga mai goyon ciki?***	
Info41	Ƙwaya 2	0.923
Info42	Ƙwaya 3	0.923
Info43	Ƙwaya 4	0.925
Info44	Maganin da ake ba wa masu goyon ciki don kariya daga cutar malariya zai	
	iya zama mai illa akan cikin da nake goyo	0.925
Info45	Ana iya shan maganin kariya daga cutar malariya ba tare da an ci abinci ba?	0.915

**Table 9:** Results of test retest reliability for motivation and behavioural skills

Item	Krippendorff’s alpha	Item	Krippendorff’s alpha
Mot1	0.981	BSkills1	0.896
Mot 2	0.965	BSkills2	0.886
Mot3	0.946	BSkills3	0.947
Mot4	0.964	BSkills4	0.890
Mot5	0.996	BSkills5	0.953
Mot6	0.981	BSkills6	0.810
Mot9	0.972	BSkills7	0.915
Mot10	0.942		
Mot11	0.953		
Mot12	0.941		

## Discussion

The results of the face validity assessment suggests that the questionnaire was comprehensible and acceptable. It also had an acceptable internal consistency, as all the Cronbach’s alpha values were within the acceptable range of 0.70 to 0.95 [20]. It also demonstrated a good reliability, with all the items of the information section having a Cohen’s kappa of greater 0.60 [21], except for one item, which was fever (*zazzabi*). However, fever being a cardinal feature of malaria, was still retained in the questionnaire, due to its relevance, but re-worded to a simpler terminology, ‘hotness of the body’ (*zafin jiki*). The Krippendorff’s alpha values for motivation, behavioural skills and ITN use were all above 0.8, and as such, acceptable [22]. Considering that Mot7 and Mot8 did not have even moderate loadings for level of pleasantness, it was reasonable to expunge them since they had high cross loadings with level of goodness, for which items with similar wordings (Mot3 and Mot4) in its category had higher factor loadings (Table 4).

Among the limitations of this study was the inadequate sample size to allow for a confirmatory factor analysis, and this should be considered in future studies.

## Conclusion

The Hausa language IMB questionnaire on malaria in pregnancy demonstrated good validity, and a high level of reliability. It is as such recommended for use among Hausa speaking communities to ensure uniformity and objectivity. It could also be translated, validated, and adapted in other malaria endemic regions. Further reliability tests like the item-response theory models should be performed to determine item difficulty and item discrimination.

## Supplementary information

**Additional file 1.**

**Additional file 2.**

**Additional file 3.**

## Data Availability

The data set, questionnaire, and consent forms for this study are available as supplementary material. To maintain respondents’ anonymity, only two indirect identifiers were retained (age and employment status).

## Abbreviations

EFA: Exploratory factor analysis
ITN: Insecticide-treated net
IPTp: Intermittent preventive treatment in pregnancy
IMB: Information-motivation-behavioural skills
KMO: Kaiser-Meyer-Olkin
WHO: World Health Organization
